# *Samotherium boissieri* from the Late Miocene of Southern Italy

**DOI:** 10.3390/life15060911

**Published:** 2025-06-04

**Authors:** Antonella Cinzia Marra

**Affiliations:** Department MIFT, University of Messina, Viale Stagno D’Alcontres 31, I-98166 Messina, Italy; antonella.marra@unime.it; Tel.: +39-090-6765452

**Keywords:** Giraffidae, Pikermian biome, central Mediterranean, *Samotherium*, *Bohlinia*, *Stegotetrabelodon*, paleogeography, paleoecology

## Abstract

*Samotherium boissieri* is a giraffid typical of the Pikermian biome, well documented at Samos and occurring in the late Miocene of the Greco-Iranian paleobioprovince. The species has been also recorded at Cessaniti in Calabria (Southern Italy), in a faunal association including other Pikermian species as well as species of Eurasian and African affinity. In this paper, Calabrian specimens are studied and compared to Samos ones. Morphological and biometrical data fall within the variability of *Samotherium boissieri* and clearly differ from the co-occurring giraffid, *Bohlinia attica*. Two partially complete forelimbs, probably referring to the same individual, permit the first full description of the *manus* bones for the species, carpals in particular. The occurrence of *Samotherium boissieri* in Calabria contributes to the wide discussion on paleobiogeographical assessments of the central Mediterranean in the late Miocene, still not well-understood.

## 1. Introduction

**Figure 1:**
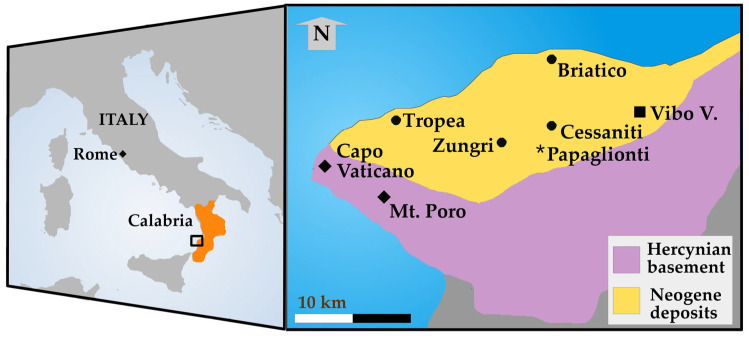
Geographic location of the fossiliferous sites and main geographic references. Square: provincial capital; dots: municipality, town; star: village; diamonds: geomorphological elements.

The late Miocene outcrops of Capo Vaticano–Monte Poro (Calabria, Southern Italy) contain a peculiar mammal fauna, with Greco-Iranian and Afro-Arabian taxa. The mammals come from a transgressive marine succession, well exposed at Cessaniti (the Province of Vibo Valentia, Calabria, Southern Italy; Figure 1). The main site is Gentile’s Quarry, near Cessaniti, where the quarrying dismantled huge quantities of sandstones and brought to light (sometimes destroyed) an impressive amount of fossil invertebrates and vertebrates [1,2]. The most iconic fossil of the whole area is the echinoid *Clypeaster*, which can be found very easily and in large quantities [3]. The fossil bones of Sirenian *Metaxytherium serresii* are also abundant, and easily recognized by the pachiostotic structure that, when broken, makes them stand out for the reddish color among the light sandstones [4,5]. Since 1993, terrestrial mammals have been found and identified at Gentile’s Quarry and correlatable sites, revealing the presence of Afro-Arabian (*Stegotetrabelodon syrticus* [6,7,8] and cf. *Certatotherium advenientis* [9]) and Greco-Iranian taxa (*Bohlinia attica* [10,11], *Tragoportax* cf. *rugosifrons* [12], and *Samotherium boissieri* [11]), besides still undetermined Bovids and an antracotheriid [2,10,11,13].

*Samotherium boissieri,* a giraffid of the Greco-Iranian bioprovince in the late Miocene from about 9–7 Ma, was replaced by its daughter species, *S. major*, characterized by larger dimensions [14]. At Samos, the former is particularly well represented [14,15], to the point that its genus name derives from the site. The occurrence of *Samotherium boissieri* confirms the presence of Greco-Iranian species in the central Mediterranean area, opening the discussion of the possible position and connection of the Cessaniti land during the late Miocene. From approximately 8.7 to 5.3 Ma, the Greco-Iranian bioprovince hosted the Pikermian biome, a characteristic association of large mammals spread in an environment where dense forests alternated with grasslands and shrubs [16,17,18]. Is the presence of Greco-Iranian species a westward expansion of the Pikermian biome sensu [17,19]? If yes, by what dispersal route, and has the land of Cessaniti had a very different geographical position in the complex geology of the central Mediterranean?

In this paper, the fossils are attributed to *Samotherium boissieri* by comparison to the wider Samos sample [14]. Moreover, they are compared to *Bohlinia attica*, also occurring in the Cessaniti mammal assemblage [10].

In addition to the scientific aspect, the occurrence of *Samotherium boissieri* in Calabria recalls an ante litteram travel from Greece to Magna Graecia. Curiously, the species is typical of Samos, one of the places where later would flourish the Ancient Greek civilization, and occurs in Calabria, part of the Magna Graecia, a wide area settled by Greek colonists from the 8th century B.C. Even more curiously, a skull of *Samotherium* seems to be figured in a Greek vase as a monster and traced back to ancient finds of fossils [19,20]. The so-called “Monster of Troy” figured on a VI century BC Corinthian vase has been identified as a *Samotherium* by the proportions of the skull with respect to humans and for the short anterior maxilla, so figured for the large nasal cohanes of the giraffid [19,20]. The “monster of Troy” has been seen as a monitor lizard by Monge-Nájera [21]. The link among myth, legend, and fossil is intriguing, and has numerous points of evidence also at Samos, where ancient Greeks identified fossil mammals as Neades or Amazons [22]. The discovery of bones resembling those of domestic animals, but with “monstrous” sizes and proportions, along with the occurrence of earthquakes, led the inhabitants of Samos to attribute them to the Neades, horrible beasts that had ravaged the island and emitted sounds so loud that they caused cracks in the ground [22]. The discovery of fossil bones, potentially including a skeletal element of *Samotherium*, in the temple of Hera in Samos is particularly interesting, as they were likely regarded as relics of the Neades [22]. The discovery of bones in the reddish sediments of Panaima at Samos has been attributed to a battle of the Amazons, who fell with their horses, likely recognized in the fossils of *Hipparion* [22]. At Capo Vaticano–Monte Poro, direct connections between fossils and myth are not documented. The fossils of *Clypeaster* enter the local folklore because they are used in fragments to make fire crackling in fireplaces. However, a local myth indicates the Greek polis of Hipparion (the current city of Vibo Valentia, Figure 1) as a place chosen by the Gods for the enchanting landscape. Due to this legend, the coast from Tropea to Briatico (Figure 1) is still named “Coast of the Gods”.

### Localities and Geological Setting

**Figure 2:**
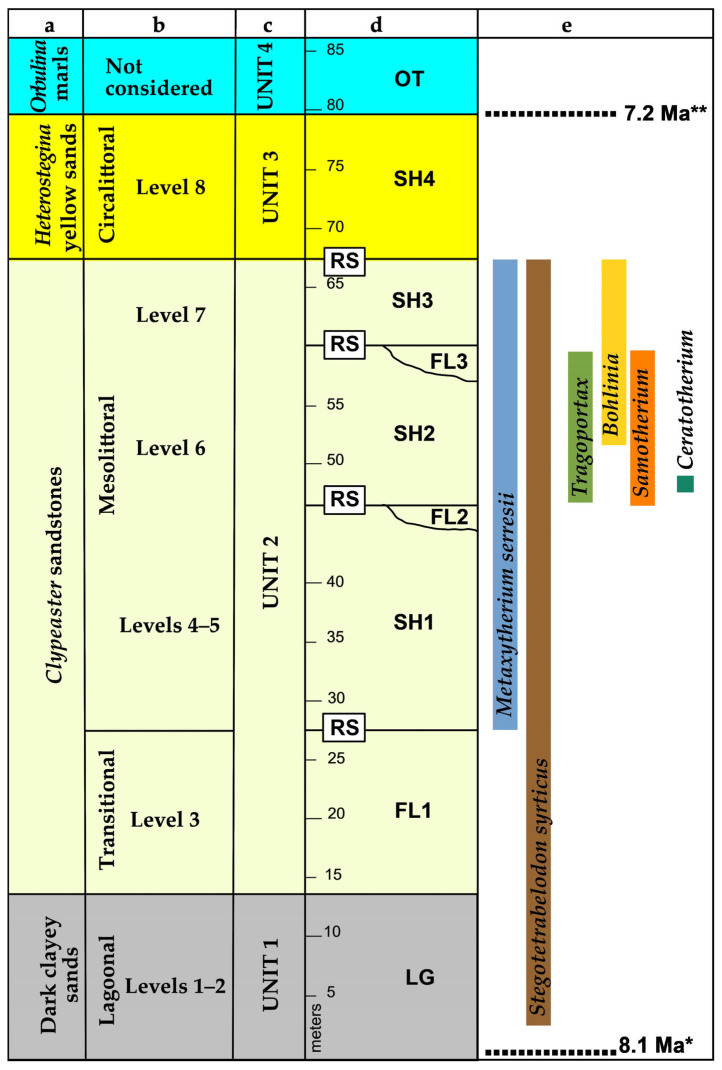
Stratigraphy of Gentile’s Quarry outcrop according to: column a, Nicotera [24]; column b, Carone and Domning [4]; column c, Gramigna [1]; column d, Marra et al. [2]. Column e: occurrences of mammals and dating based on magnetostratigraphy (*) and nannoplankton biozones (**) according to Marra et al. [2]. LG: lagoonal deposits; FL: fluvial deposits; SH: shoreface deposits; OT: offshore transition; RS: ravinement surface.

**Figure 3:**
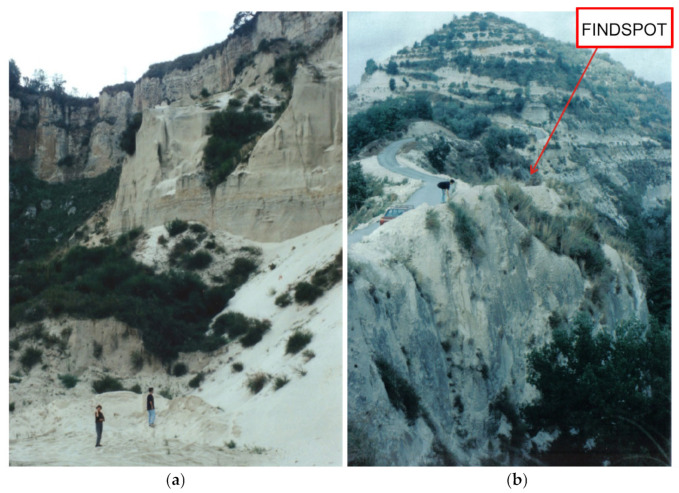
The abandoned quarry at Malopara: (**a**) overview; (**b**) level with *Samotherium*.

The studied fossils come from an abandoned quarry at Malopara (the municipality of Zungri, the Province of Vibo Valentia, Calabria, Southern Italy; Figure 1) and from Gentile’s Quarry (the municipality of Cessaniti, the Province of Vibo Valentia, Calabria, Southern Italy; Figure 1, Figure 2 and Figure 3). The sites are included in the Capo Vaticano–Monte Poro area, where a Neogene succession rests on a Paleozoic crystalline substratum from the inland to the coast [1,2,4,23] (Figure 1 and Figure 2).

The most representative succession of the area is well exposed at Gentile’s Quarry (Cessaniti), which produced the main part of the fossil mammals, bought to light by quarrying works and collected by amateur paleontologists [24]. The succession, schematized in Figure 2 according to different authors [1,2,23,25], represents a marine transgression, with some intervals represented by fluvial deposits (FL in Figure 2) followed by ravinement surfaces (RS in Figure 2; intended as erosional surfaces formed during transgressions), probably occurring under tectonic control. In proximity to the ravinement surfaces, bone beds of *Metaxytherium* and other mammals are found [1]. The succession has an age between 8.1 and 7.2 Ma [2]. In about 1 Ma, the transgression changed the environmental conditions at Cessaniti, from marginal lagoons protected by sandy barriers to a shallow sea up to marine conditions [1,2,26,27,28].

The specimens studied in this paper come from the upper part of the “*Clypeaster* sandstones” outcropping throughout the area of Cessaniti–Zungri with different thickness and characterized by the impressive quantity of echinoid shells and sirenian bones, attributed to *Metaxytherium serresii*. From the *Clypeaster* sandstones come a rich land mammal assemblage (Figure 2, column e), including *Bohlinia attica* [2,10,11], *Tragoportax* cf. *rugosifrons* [12], *Stegotetrabelodon syrticus* [6,7,8], cf. *Ceratotherium advenientis* [9], still undetermined Bovids and Anthracotheriids [2,11,13], and *Samotherium boissieri* studied in this paper.

The taphonomy of the site is not fully clear because the fossils have not been collected through systematic excavation (see Section 2, Materials and Methods). A large part of mammal remains present abrasion and damage by river transport, while many also show incrustations by marine organisms. It is quite evident that the mammal remains reached the sea carried by rivers to the seabed before final burial, providing a substratum to encrusting marine organisms. At Malopara (Zungri; Figure 1 and Figure 3), two forelimbs, probably belonging to the same individual of *S. boissieri*, have been found from levels correlatable to upper SH1-SH2 (Figure 2 column d) of the *Clypeaster* sandstones. As reported by the collectors, the bones were slightly scattered at close range in two groups: one including left radius, carpals, and metacarpal; one including some right carpals, sesamoids, metacarpal, and phalanges. A fragmentary humerus is too damaged to be included in this study. Some bones are altered by weathering; many are slightly abraded and some present encrustations by marine organisms. It can be assumed that the carcass was transported by a river still with connective tissues, maybe during a flood event, and then laid down on the seabed in partial anatomical connection. There is also the possibility that the animal died near the sea. Research to find other skeletal elements has not been successful. The find is unique for its completeness.

At Gentile’s Quarry, two metacarpals and an astragalus of *S. boissieri* come from the level SH2 of the *Clypeaster* sandstones.

## 2. Materials and Methods

The materials studied are in Table 1, with the locality and the inventory number attributed by the institution where the fossils are exhibited [24,29]: MuRi is for the City Museum of Ricadi (Vibo Valentia) and SiMU is for the Museal System of the University of Calabria (Cosenza). An astragalus without inventory, studied in 2010, is included in this paper. The astragalus was collected in the “*Clypeaster* sands” at Cava Gentile by the late Mario Bagnato and stored at Parghelia (VV) in the headquarters of the “Gruppo Paleontologico Tropeano” (GPT), a former amateur association. The hall and storage rooms were later integrated into the “Museo della Memoria” (MuMe) in Parghelia; however, the astragalus is currently unavailable, and an inventory check has been requested.

The measurements have been taken with a manual caliper, following the standards of Von Den Driesch [30] adapted for the definition of Kostopoulos [14] to render clear the comparisons. The RI (Robustness Index) is indicative of metapodial slenderness and is calculated as the transverse diameter of the shaft/the length of the shaft.

The fossils have been compared to the other occurring giraffid, *Bohlinia attica* (Table 1). The anatomical terminology adopted is from Bohlin [31], Kostopoulos [32,33] Rios et al. [34], and Solounias and Danowitz [35].

The collecting methods have not been systematic for the quarrying and for the great dispersion of the fossils. Fossils were collected through the ongoing efforts of amateur paleontologists, which gathered emerging fossils during breaks in quarrying activities at Gentile’s Quarry and nearby areas, particularly after rainfall, noting the site and altitude of each find, though not always adhering to strict scientific protocols [24]. The collectors referenced Nicotera’s informal stratigraphy (column “a” in Figure 2, [23]) and recorded specific conditions useful to track the finds in the stratigraphic column [24]. This approach allowed them to retrieve many fossils that would otherwise be destroyed and that would not be systematically excavated due to their low concentration in the sediment. This collection method yielded a rich fossil record [24,29], but compromised the taphonomic analysis.

## 3. Systematic Paleontology

Class: Mammalia Linnaeus, 1758;

Order: Artiodactyla Owen, 1848;

Suborder: Ruminantia Scopoli, 1777;

Family: Giraffidae Gray, 1821;

Genus: *Samotherium* Forsyth-Major, 1888;

Species: *Samotherium boissieri* Forsyth-Major, 1888;

Studied materials are listed in Table 1.

Stratigraphic occurrence: Late Miocene (Upper Tortonian; European Land Mammal Age: Turolian) of Cessaniti and Zungri (Vibo Valentia, Italy), *Clypeaster* sandstones (levels 6 and 7 according to Carone and Domning [4], corresponding to SH2-SH3 in Marra et al.’s study [2]).

### 3.1. Descriptions

#### Forelimbs

**Figure 4:**
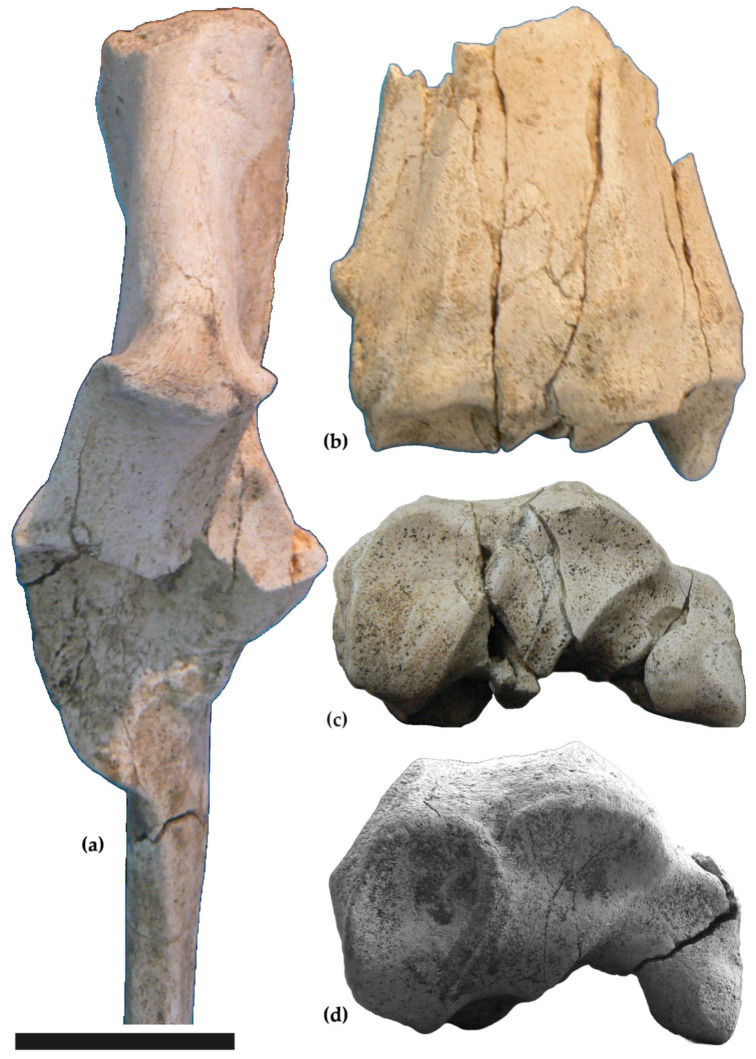
*Samotherium boissieri* from Malopara: left proximal ulna (MuRi-870) in frontal view (**a**); left distal radius (MuRi 895) in dorsal (**b**) and distal (**c**) views; *Bohlinia attica* from Cessaniti: (**d**) left distal radius in distal view (MuRi-874). Scale bar: 5 cm.

Two partial forelimbs attributable to the same individual come from Malopara. The left forelimb includes distal radius (MuRi-895), proximal ulna (MuRi-870; Figure 4a), all carpals (MuRi878, 880,879,881,882,883), and metacarpal (MuRi-865). The right forelimb includes some carpals (MuRi 884, 885, 886), metacarpal (MuRi-864), first phalanx of the III toe (MuRi-888), first phalanx of the IV digit (MuRi-887), second phalanx of the III digit (MuRi-890), second phalanx of the IV digit (MuRi-889), third phalanx of the III digit (MuRi-892), and third phalanx of the IV digit (MuRi-891).

In the preserved proximal ulna (MuRi-870, Figure 4a), the posterior margin of the olecranon is straight. The ulnar notch is curved and wide. Measurements: depth across the anconeal process = 100.4 mm; smallest breadth of the olecranon = 85; breadth across the coronoid process = 56.4.

The two distal radii (MuRi-866 and MuRi-895, Figure 4b,c) are fragmented. The styloid process is prominent. The groove for the extensor carpi radialis and the groove for the common extensor tendon are well evident. The ulnar notch is prominent. Despite fragmentation, it is possible to describe the distal articular surface, which presents oblique facets for the scaphoid and the intermedium, and a deep facet for the ulnare is deep, subvertical in the margin with the facet for the intermedium. The fragmentation of the bones makes reliable measurements difficult; the approximate distal breadth of MuRi-895 is 96.4 mm.

**Figure 5:**
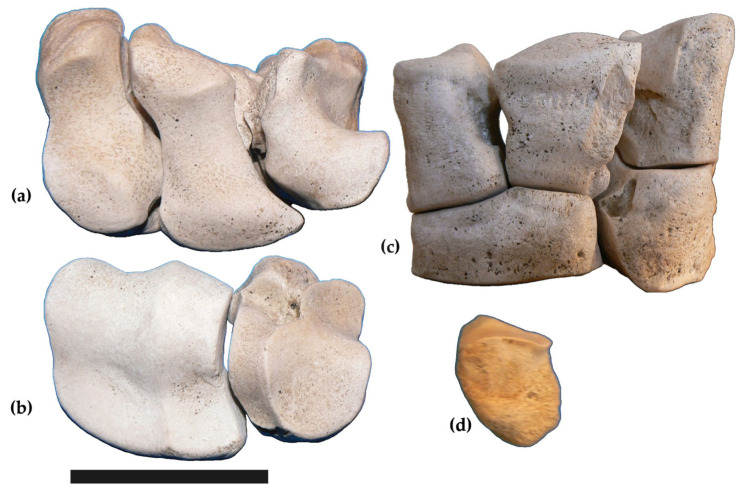
*Samotherium boissieri* from Malopara. Carpals in anatomical connection: (**a**) proximal row of left carpals in proximal view (scaphoid MuRi-878, intermedium MuRi-879 and ulnare MuRi-880); (**b**) distal row of left carpals in proximal view (magnotrapezoid MuRi-882, uncinatum MuRi-881); (**c**) carpals in anatomical connection in anterior view; (**d**) pisiform MuRi-883. Scale bar: 5 cm.

Carpal bones belonging to the same individual of *Samotherium boissieri* are here described in their entirety for the first time. In the absence of comparison specimens for most of them, they can be attributed to the species by association with the metacarpals, which clearly have the characters of *S. boissieri*. The bones are figured in anatomical connection (Figure 5), but were found slightly scattered.

Scaphoid (MuRi-878, left, Figure 5a,c). Proximal view: the articular surface enlarges towards the anterior side; it is high and convex posteriorly, proceeds uniformly concave, and drops down anteriorly. Lateral view: the proximal articular surface continues laterally, where forms an anteroposterior elongated facet for the semilunar; on the lateral side a quadrangular facet for the semilunar is present distally. Medial view: the bone is flat and rough, without facets. Distal view: the facet for the magnotrapezoid is irregularly rectangular. The posterior side of the bone presents a protruding tuberosity. Greatest breadth (MuRi-878): 30.6 mm.

Intermedium (MuRi-879, left, Figure 5a,c; MuRi-885, right). Proximal view: the articular surface is narrow, convex and elevated posteriorly, narrower in the middle, where it is concave, and convex, elevated and wide anteriorly. Lateral view: an irregularly squared facet is proximally for the ulnare; a sub-oval facet extends upwards from the distal articular surface, in a central-posterior position. Medial view: in the upper part, an elongated facet is present for the scaphoid; another facet for the scaphoid is located anteriorly, near the distal margin. Distal view: the articular surface for the magnotrapezoid is convex anteriorly and concave posteriorly. Greatest breadth of MuRi-879: 31 mm; MuRi-885: 32 mm.

Ulnare (MuRi-880, left, Figure 5a,c; MuRi-884, right). Proximal view: the articular surface is high and convex posteriorly and drops down anteriorly, where it is concave; it is separated by a rim in a wider medial portion and a narrower lateral portion. Lateral view: two facets for the uncinatum are present, a quadrangular one anteriorly and a semicircular one posteriorly. Medial view: the bone is rough and has no facet. Distal view: the articular surface for the metacarpal is flat, quadrangular, and wider anteriorly. Greatest breadth of MuRi-880: 30 mm; MuRi-884: 29 mm.

Magnotrapezoid (Muri-882, left, Figure 5b,c). Proximal view: the articular surface is high and convex posteriorly and drops down anteriorly, where it is concave; it is separated by an antero-posterior rim in a wider medial articulation and a narrower lateral articulation. Lateral view: the articulation for the uncinatum consists of an anterior quadrangular facet and a posterior semicircular facet. In the medial view, the bone is flat, without facets. Distal view: a flat and quadrangular facet for the metacarpal is present. Greatest breadth of MuRi-882: 42 mm.

Uncinatum (Muri-881, left, Figure 5b,c; Muri-886, right). Proximal view: the articular surface is higher and larger anteriorly and extends in the posterior side; it is separated by a rim in a medial convex facet and a lateral concave facet. Lateral view: facets not present. Medial view: a semicircular articular facet extends around a shallow depression. Posterior view: the upper part is occupied by the extension of the proximal surface. Distal view: a flat articular surface is for the metacarpal. Greatest breadth of MuRi-881: 36.7; MuRi-886: 36.6.

Pisiform. The pisiform (MuRi-883, left; Figure 5d) is robust with an elongated facet for the ulnare. Greatest breadth: 27.7.

**Figure 6:**
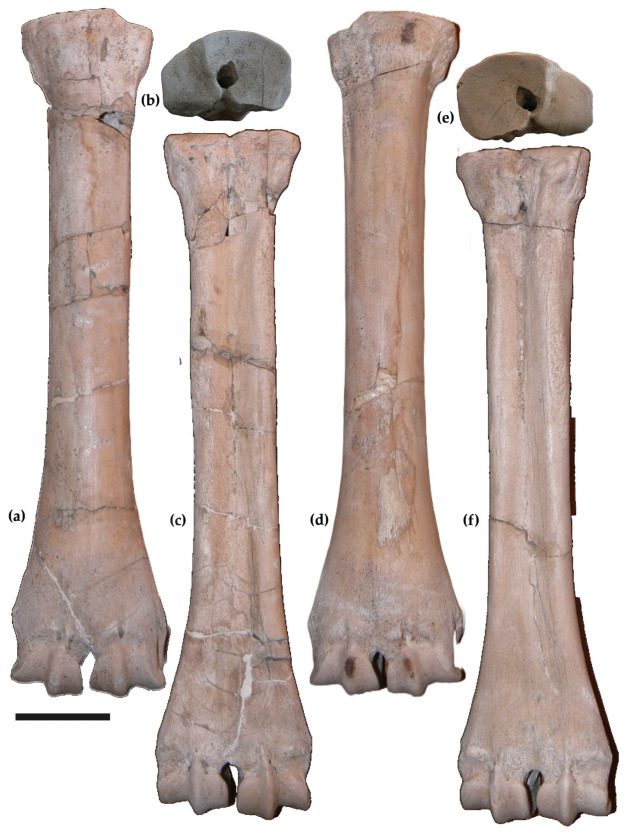
*Samotherium boissieri* from Malopara. Left metacarpal (MuRi-865) in dorsal (**a**), proximal (**b**), and ventral (**c**) views. Right metacarpal (MuRi-864) in dorsal (**d**), proximal (**e**), and ventral (**f**) views. Scale bar: 5 cm.

The left (MuRi-865, Figure 6a–c) and the right (MuRi-864, Figure 6d,e) metacarpals have a slender diaphysis, with a RI = 11.8 (MuRi-864) and 11.7 (MuRi-865). The synovial fossa is closed. The proximal articulation has a semicircular profile, and the postero-medial and palmar tubercles are absent. The proximal articular surface has a flat rectangular facet for the capitatum, more elongated in the lateral direction. It is separated by a step from the flat squared facet for the uncinatum, lying on a lower plane. In the posterior view, the shaft has a shallow sulcus, delimitated by wide and smoothed crests.

The measurements of the metacarpals (MuRi-864, MuRi 865, and MuRi-875, Table 2) fall in the variability of *S. boissieri*, and are sensibly smaller than *Bohlinia* from Cessaniti (MuRi-972, Table 2).

The sesamoids are characterized by elongated articular facets.

**Figure 7:**
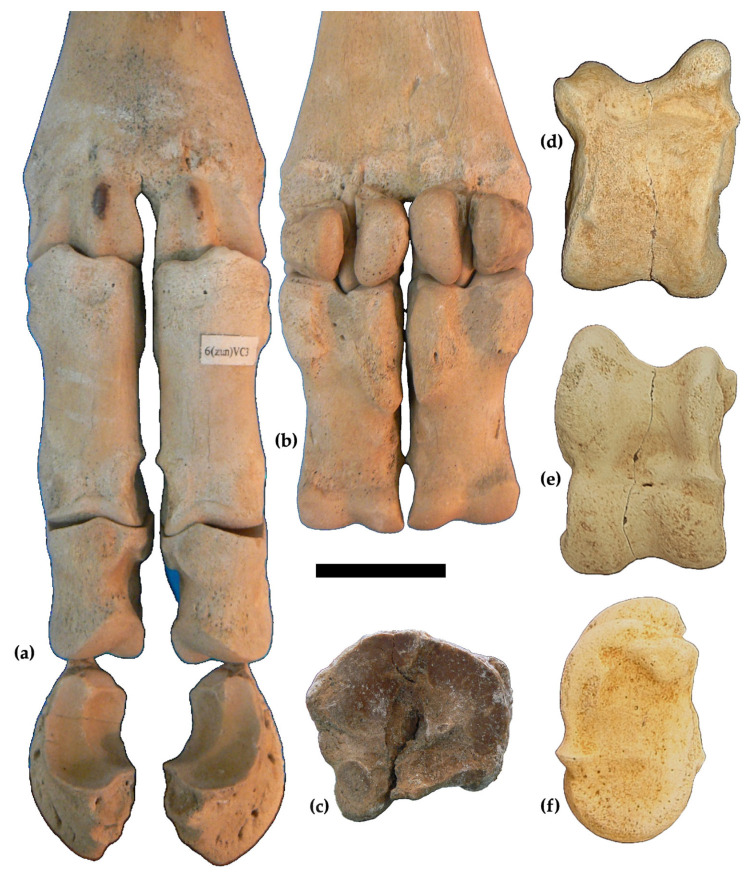
*Samotherium boissieri*. Right phalanges (MuRi-888, 887, 890, 889, 892, 891) in anatomical connection with the metacarpal (MuRi-864) in dorsal view (**a**) and detail of the ventral view with sesamoids (**b**), from Malopara. Left metatarsal (SiMU-CMA07) in proximal view (**c**); right astragalus (GPT-MuMe) in ventral (**d**), dorsal (**e**), and medial (**f**) views; from Cessaniti. Scale bar: 5 cm.

The proximal phalanges (MuRi-888, MuRi-887; Figure 7a,b; Table 3) belonging to the right manus are slender, larger proximally than distally. The intermediate phalanges are short (MuRi-880, MuRi-889; Figure 7a; Table 3). The distal phalanges (MuRi-892, MuRi-891, Figure 7a; Table 3) have very concave facets; the external one is larger than the internal one.

The astragalus (GPT-MuMe, Figure 7d–f) is massive. Dorsal view: the lateral ridge of the trochlea is more elevated and slightly larger than the medial one; the medial ridge of the trochlea protrudes with a tuberosity toward the medial side; the groove of the trochlea is U-shaped; the central fossa is shallow; the head has a lateral articulation elongated in antero-posterior direction; the medial bulge of the collum talii is located almost at 1/3 of the medial margin. Ventral view: the proximal triangular fossa is deep and laterally extended until the interarticular groove; the medial scala is well evident. Medial length: 80.0 mm; lateral length: 90 mm; depth of lateral half: 48; depth of medial half: 54; distal transverse diameter: 58.

The metatarsals (SiMU-CMA06 and SiMU-CMA07, Table 4; Figure 7c) have a robust shaft furrowed by a wide posterior groove delimited by two crests located near the medial and the lateral sides. In the proximal epiphysis, the facet for the naviculo-cuboid is slightly convex towards the center, flat externally. The lateral facet for the cuneiform is C-shaped, wide, and flat, slightly convex in its posterior portion. The medial facet for the cuneiform is rectangular with rounded corners.

### 3.2. Comparisons

The comparisons are mainly addressed to the large sample of *Samotherium boissieri* from Samos, Greece, as well as to comparative studies and descriptions [14,15,34,35,36,37,38]. Radii, metacarpals, and astragalus are also compared to the specimens of *Bohlinia attica* from Cessaniti [10].

The two forelimbs found at Malopara include radii and metacarpal bones; the latter are very significant for taxonomy and can be considered crucial to attributing the two *mani* found at Malopara.

The two distal radii (MuRi-895, Figure 4b,c, and MuRi-866) are fragmentary and can be attributed to *S. boissieri* for the general morphology and the available measurements. Compared to the radius of *B. attica* from Cessaniti (Muri-874), the distal articular surface of MuRi-895 has shallower facets for the scaphoid and the intermedium and a deeper facet for the ulnare.

**Figure 8:**
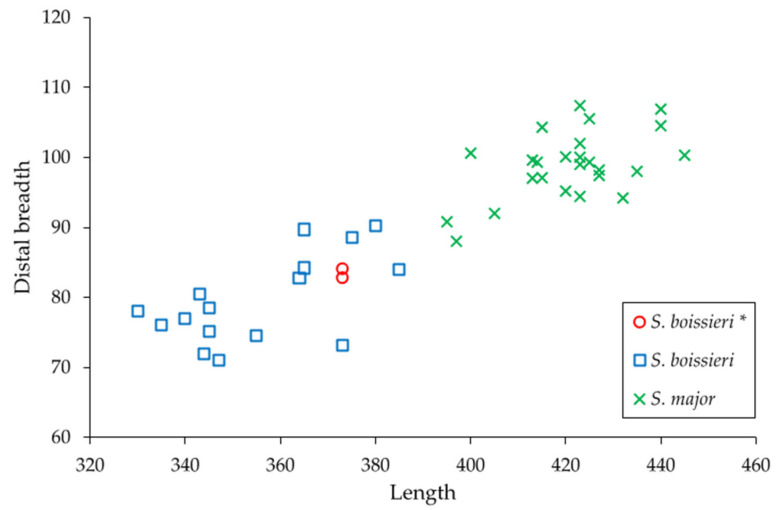
Dispersion plot of the length/distal breadth (transverse diameter) of the metacarpals of *Samotherium boissieri* (squares) and *Samotherium major* (crosses) from Samos [14] and *Samotherium boissieri* from Malopara (circles, marked with *).

In the metacarpals (MuRi-864, MuRi-965; Figure 6), the semicircular profile and the squared facets of the distal articular surface as well as the shallow posterior sulcus of diaphysis are similar to *S. boissieri* [14,15,34], but differ from the semioval profile of the proximal articular epiphysis, the oval proximal articular facets, and the deep posterior sulcus observed in the right metacarpal of *B. attica* from Cessaniti (Mu.Ri-972; Figure 6; [10]). The measurements and the bone proportions calculated in the dispersion plot (Figure 8) fall within the variability known for *Samotherium boissieri* [14]. The two metacarpals probably belonged to male individuals, by comparison with the range of sexual bimodality [14]. The robustness indices (RI: Transverse diameter of shaft/length; 11.8 in MuRi-864, 11.7 in MuRi-865) fall within the variability known for *Samotherium boissieri* from the Mytilinii Basin of Samos (from 10.4 to 13.6, mean: 12.45, calculated on 12 specimens; [14]) and differs from the variability known for *B. attica* (5.56–8.57; mean value 7.3 calculated on 11 specimens; [34,39,40,41]).

**Figure 9:**
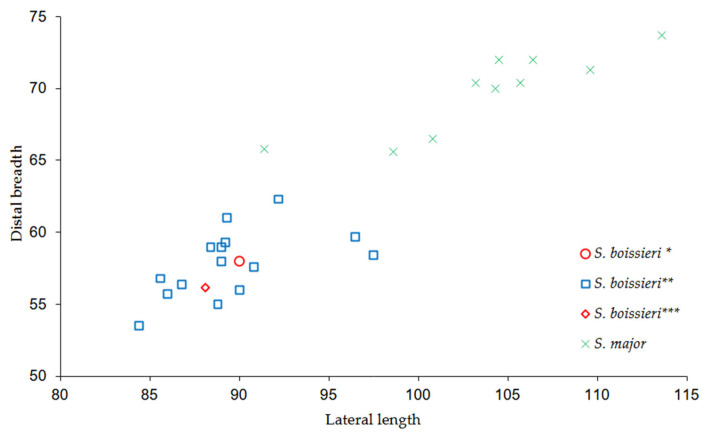
Dispersion plot of the lateral length/distal breadth (transverse diameter) of the astragalus of *Samotherium boissieri* (squares; marked with two *) and *Samotherium major* (crosses) from Samos (measurements after K 2009 [14]), *Samotherium boissieri* (diamond; marked with three *) from Kavakdere [37], and *Samotherium boissieri* from Malopara (circles; marked with one *).

The astragalus (GPT-MuMe) falls within the biometrical variability of *Samotherium boissieri* (Figure 7d–f and Figure 9; Table 5). It is worth noting that *Bohlinia attica* from Cessaniti and other sites [35,36,37,39] also has similar measurements. The marked differences between *Samotherium boissieri* and *Bohlinia attica* astragali are in the morphology, as also observable in the specimens from Cessaniti referred to the two species: the lateral ridge of the throclea is less elevated and robust in *Samotherium* than in *Bohlinia*; the groove of the trochlea is more marked than in *Bohlinia*, where it is wide; the medial bulge of the *collum talii* is located more posteriorly in *Bohlinia*; in antero-posterior direction, the head is larger in *Samotherium* than in *Bohlinia*; the proximal triangular fossa is less extended laterally in *Bohlinia*; the medial scala is more evident in *Samotherium*. Kostopoulos [14] indicated sexual dimorphism ranges expressed by a bimodality also observable in Figure 9; the astragalus from Cessaniti falls within male variability.

**Figure 10:**
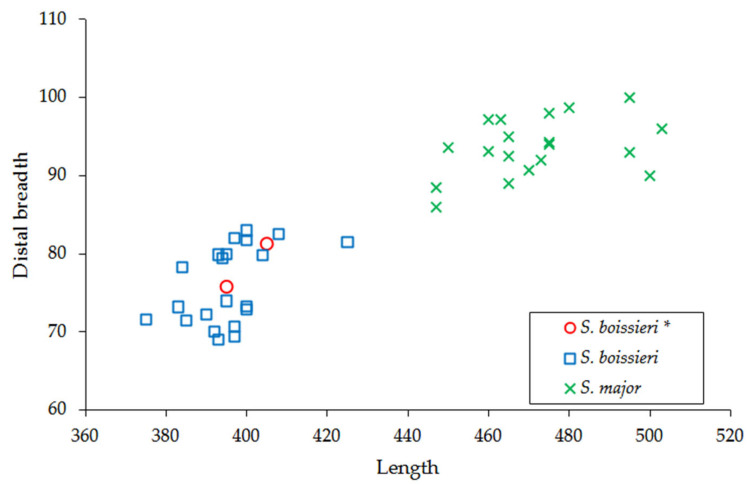
Dispersion plot of the length/distal breadth (transverse diameter) of the metatarsals of *Samotherium boissieri* (squares) and *Samotherium major* (crosses) from Samos [14] and *Samotherium boissieri* from Malopara (circles; marked with *).

The two metatarsals fall within the morphological and biometrical variability of the species *Samotherium boissieri* (Figure 7c and Figure 10) and are considerably shorter than *Bohlinia attica* [39,40]. The robustness indices (RIs) are 10.6 and 10.4, within the variability of *Samotherium boissieri* from Samos (9.5–12.5; mean = 10.7 calculated in 20 specimens [14]). According to the sexual bimodality indicated by Kostopoulos [14], the specimen SiMu-CMA06 falls next to the female size range, while SiMu-CMA06 falls near the male range.

## 4. Discussion

*Samotherium boissieri* is a species spread across the Greco-Iranian bioprovince, from 8.9 to about 7 Ma (https://nowdatabase.org/ accessed on 4 May 2025); its occurrence at Cessaniti needs broad discussion on biochronology, paleoecology and paleogeography of the whole mammal association, represented by a significant number of fossils (Figure 2, Table 6 and Table 7).

### 4.1. Biochonology and Distribution

The Artiodactyls occurring at Cessaniti are *Samotherium boissieri, Bohlinia attica*, and *Tragoportax* cf. *rugosifrons*. The three species are associated only at Cessaniti, while *Samotherium boissieri* co-occurs with *Bohlinia attica* at Corakyerler (Turkey) and Injana (Iraq) and with *Tragoportax rugosifrons* at Samos (Greece) and Taraklia (Moldova); *Bohlinia attica* and *Tragoportax rugosifrons* co-occur in the Bulgarian sites of Hadjidimovo and Strumyani, and at Karaslari in the Republic of Macedonia (data from https://nowdatabase.org/now/database/, accessed on 3 May 2025; Table 7). The biochronologic distribution of the three species in the Greco-Iranian bioprovince is coherent with the age of Cessaniti (Table 7).

The species *Stegotetrabelodon syrticus* is only known from the site of Sahabi, dated 6.7 Ma [43] (https://nowdatabase.org/ accessed 3 May 2025). However, until few years ago, the species included Arabian specimens, recently enriched by new finds and attributed to *Stegotetrabelodon emiratus,* bracketed in a time interval of 8–6 Ma [44,45]. The studies on *Stegotetrabelodon* from Cessaniti, carried out in 1993 [6] and 2016 [7], considered the whole variability of *Stegotetrabelodon syrticus*, including the Arabian specimens now called *Stegotetrabelodon emiratus*. Some plesiomorphic characters were inferred on the sample from Cessaniti. A revision of the proboscideans from Cessaniti, also including recent new finds, is in progress. Giraffidae are signaled in the Baynunah Formation (United Arab Emirates): some are indetermined, other are dubiously considered *Palaeotragus*/*Samotherium* [42,46], and a partial skeleton is attributed to *Palaeotragus* aff. *germaini* [47].

The new rhinocerotid, cf. *Ceratotherium advenientis,* is not biochronologically informative given its uncertain systematic position, probably next to the African genera *Diceros* and *Ceratotherium* [9].

### 4.2. Paleoecology

*Samotherium boissieri* and *Bohlinia attica* are both considered browsers by Solounias et al. [17,48]. Rios et al. consider *Samotherium boissieri* a seasonal mixed feeder and *Bohlinia attica* a grazer [49]. *Tragoportax rugosifrons* was a grazer and the genus *Tragoportax* probably occupied an ecological niche like that of the modern *Hippotragus*, consisting of forest–savannah environments interspersed with open spaces [17,50]. *Stegotetrabelodon* is considered a ground-dwelling grazer–browser and *Ceratotherium* a ground-dwelling grazer. These palaeoecological characteristics fall in the variability of the Pikermian biome, typical of the Greco-Iranian bioprovince and described as a sclerophyllous evergreen woodland interspersed with grassy meadows, inhabited by a uniform mammal fauna [16,17,51,52,53]. The uniformity of the Pikermian biome led to the consideration of the fauna as a “chronofauna” in the sense of Olson [51]. However, Kostopoulos [18] challenged this notion, pointing out variations in species diversity and biochronology across different sites. In contrast, Eronen et al. [54] applied the Genus-level Faunal Resemblance Indices (GFRIs) to several locations, finding a resemblance degree exceeding 50%. The Pikermian biome emerged approximately 8.7 million years ago, reaching its peak around 7 million years ago, and eventually disappeared around 5.3 million years ago, likely due to increasing seasonality and climatic changes linked to oceanic water circulation in the North Atlantic [16,18,55]. Pikermian species have also been reported outside the Greco-Iranian bioprovince, with occurrences from China to Spain and limited finds in Africa [51,55,56,57,58].

The extension of the Pikermian biome at Cessaniti opens intriguing considerations, strictly related to paleogeography.

### 4.3. Paleobiogeography

The central Mediterranean paleogeography is difficult to reconstruct for the complex geology of the area. Cessaniti is in the Capo Vaticano–Monte Poro area, between the Calabrian Arc and the Tyrrhenian Basin. The Capo Vaticano promontory has been separated from the Serre massif by the opening of the Mesima graben during the Miocene–Pleistocene and has been strongly affected by Quaternary tectonics [59,60]. The transgressive event of the late Miocene affected a land whose extension and position are not known. The Cessaniti mammal assemblage is indicative of a land having relationships with the Greco-Iranian and possibly the Afro-Arabian bioprovinces. This land had to be connected extensively enough to support large mammals with specific ecological needs. The Cessaniti land was not isolated, as the absence of endemic characteristics in mammals indicates, and had not had faunal exchanges nor territorial continuity with the two bio-provinces identified in Italy (Tusco-Sardinia and Apulo-Abruzzi) and the site of Gravitelli in Sicily:

- The fossil record from the Tusco-Sardinian bioprovince is notably endemic and impoverished, largely due to a prolonged period of insular conditions [61,62,63,64].
- The Apulo-Abruzzi area featured strongly endemic mammals since the early Tortonian [61,65,66,67,68].
- The Gravitelli mammal assemblage (Messina, Sicily) is based on fossils no longer available to the study, because they were destroyed during the earthquake that hit the city of Messina in 1908. The revision of available museum casts and the papers by Seguenza [69,70] revealed a non-endemic fauna with European affinity and possibly two African taxa [71,72]. The re-consideration of the stratigraphy described by Seguenza could indicate an age more ancient than the Messinian age originally attributed, maybe older than 7 Ma [71]. The site is no longer investigable for the intense urbanization. The mammal association does not have taxa in common with Cessaniti, except for *Ceratotherium* sp.

The paleogeographic scenarios for the Cessaniti land are as follows:

- Land connected to North Africa: in this case, the Greco-Iranian taxa come from an expansion to Africa of the Pikermian biome; this widely hypothesized condition is not strongly supported by evidence.
- Land connected to Eastern Europe: in this case, the land was subject to complex movements, maybe related to the Adria plate; this hypothesis is not supported by geological data.

## 5. Conclusions

The attribution of the giraffid remains here ascribed to *Samotherium boissieri*, in addition to *Bohlinia attica* and *Tragoportax* cf. *rugosifrons*, confirms the similarities of the Cessaniti faunal assemblage to the Greco-Iranian bioprovince.

The presence of *Ceratotherium* and *Stegotetrabelodon* would suggest an Afro-Arabian component in the assemblage. However, the presence of a *Stegotetrabelodon syrticus* with plesiomorphic characters leads to a revision because the specimens from Cessaniti have been compared to the whole variability of the species, which included the Arabian specimens recently attributed to *Stegotetrabelodon emiratus*.

The mammal assemblage of Cessaniti reveals an area without connection to other central Mediterranean lands. The original position and connections of the land of Cessaniti in the Mediterranean during the late Miocene is still unknown.

## Figures and Tables

**Table 1:** Giraffidae from Cessaniti described and compared in this paper.

Specimen	Locality	Inventory Number
*Samotherium boissieri*		
Ulna—left proximal epiphysis	Malopara	MuRi-870
Radius—left distal epiphysis	Malopara	MuRi-895
Radius—right distal epiphysis	Malopara	MuRi-866
Scaphoid—left	Malopara	MuRi-878
Intermedium—left	Malopara	MuRi-879
Intermedium—right	Malopara	MuRi-885
Ulnare—left	Malopara	MuRi-880
Ulnare—right	Malopara	MuRi-884
Magnotrapezoid—left	Malopara	MuRi-882
Unicinatum—left	Malopara	MuRi-881
Unicinatum—right	Malopara	MuRi-886
Sesamoids and pisiform	Malopara	MuRi-883
Metacarpal—left	Malopara	MuRi-865
Metacarpal—right	Malopara	MuRi-864
Metacarpal. distal end—left	Cessaniti	MuRi-875
First phalanx, III digit—right	Malopara	MuRi-888
First phalanx, IV digit—right	Malopara	MuRi-887
Second phalanx, III digit—right	Malopara	MuRi-890
Second phalanx, IV digit—right	Malopara	MuRi-899
Third phalanx, III digit—right	Malopara	MuRi-892
Third phalanx, IV digit—right	Malopara	MuRi-891
Third phalanges III and IV digit	Cessaniti ?	MuRi-894
Astragalus—right	Cessaniti	? GPT-MuMe
Metatarsal—left	Cessaniti	SiMU-CMA07
Metatarsal—right	Cessaniti	SiMU-CMA06
*Bohlinia attica*		
Radius—left	Cessaniti	MuRi-874
Metacarpal—left	Cessaniti	MuRi-972
Astragalus—left	Cessaniti	SiMU-CMA02
Astragalus—right	Cessaniti	Muri-877

Uncertain provenance or storage are marked with “?”.

**Table 2:** Measurements of the metacarpals. L: length; TDp: transverse diameter of proximal epiphysis; APDp: antero-posterior diameter of proximal epiphysis; TDdia: transverse diameter of diaphysis; CD: circumference of diaphysis; APDdia: antero-posterior diameter of diaphysis; TDd: transverse diameter of distal epiphysis; APDd: antero-posterior diameter of distal epiphysis.

Inventory	L	TDp	APDp	TDdia	CD	APDdia	TDd	APDd
MuRi-865	373	75.7	48.2	43.7	136.5	36.5	84.1	48.2
MuRi-864	373	74.4	49.2	44	136.5	36.3	82.8	48.0
MuRi-875	-	-	-	-	-	-	83.4	48.0
MuRi-972	-	90.8	53.1	-	-	-	88.0	49.9

**Table 3:** Measurements of the phalanges of *Samotherium boissieri* from Zungri. L: length; TDp: transverse diameter of proximal epiphysis; SD: smallest breadth of diaphysis; TDd: transverse diameter of distal epiphysis; DLS: greatest diagonal length of the sole; Ld: length of dorsal surface; MBs: middle breadth of the sole.

Specimen	Inventory	L	TDp	SD	TDd
First phalanx, III digit	MuRi-888	100.9	41.1	32.9	32.2
First phalanx, IV digit	MuRi-887	98.0	40.1	32.7	31.4
Second phalanx, III digit	MuRi-890	65.2	37.7	30.6	32.2
Second phalanx, IV digit	MuRi-899	61.3	36.9	30.9	31.0
		**DLS**	**Ld**	**MBs**	
Third phalanx, III digit	MuRi-892	80.7	59.1	30.5	
Third phalanx, IV digit	MuRi-891	80.5	61.7	29.3	

**Table 4:** Measurements of the metatarsals of *Samoherium boissieri* from Cessaniti. L: length; TDp: transverse diameter of proximal epiphysis; APDp: antero-posterior diameter of proximal epiphysis; TDdia: transverse diameter of diaphysis; CD: circumference of diaphysis; APDdia: antero-posterior diameter of diaphysis; TDd: transverse diameter of distal epiphysis; APDd: antero-posterior diameter of distal epiphysis.

Inventory	L	TDp	APDp	TDdia	CD	APDdia	TDd	APDd
SiMU-CMA06	395	69.9	65.9	41.0	15.0	33.6	75.8	46.0
SiMU-CMA07	405.0	78.0	72.0	43.0	15.5	37.0	91.3	50.3

**Table 5:** Measurements of astragalus of *Samotherium boissieri* and *Bohlinia attica*. Llat: lateral length; Lmed: Medial length; TD: distal transverse diameter; APDd: antero-posterior diameter of distal epiphysis.

Species	Ref.	Llat	Lmed	TD	APDd
Cessaniti specimen		90	80	58	54
*Samotherium boissieri*					
	[14]	90.08	78.5	57.6	
	[14]	96.5	82.3	59.7	
	[14]	97.5	85.1	58.4	
	[14]	89.2	78.6	59.3	
	[14]	89.0	79.2	58.0	
	[14]	88.4	78.0	59	
	[14]	86.8	73.7	56.4	
	[14]	85.6	75.0	56.8	
	[14]	89.3	76.2	61.0	
	[14]	84.4	76.5	53.5	
	[14]	88.8	77.6	55.0	
	[14]	89.0	75.8	59.0	
	[14]	86.0	77.2	55.7	
	[14]	90.0	76.6	56.0	
	[14]	92.2	81.0	62.3	
	[37]	88.1	80.95	56.16	
*Bohlinia attica*					
	[10]	89	78.6	59	55.6
	[10]		81	59	
	[36]	87.7–103.4 (min–max)	76.5–89.6 (min–max)	55–68 (min–max)	46.5–61.1 (min–max)
	[36]	103	99	76.6	
	[36]	105	94	70	
	[36]	105	91	71	
	[36]	108	94	73	
	[42]	83.8			59.3
	[42]		87.6		
	[39]		81	59	

**Table 6:** List of fossils of the land mammals of Cessaniti from Gentile’s Quarry and correlatable deposits at Papaglionti (*) and Malopara (**).

	*Stegotetrabelodon* *syrticus*	cf. *Ceratotherium* *advenientis*	*Tragoportax* cf. *rugosifrons*	*Bohlinia* *attica*	*Samotherium* *boissieri*
Unit 2	mandible; incisor; fragmentary molar; two fragmentary humeri *; right II metacarpal; incomplete femur	fragmentary skull; two fragmentary teeth; few postcranial bones	hemimandible; radius; astragalus; anterior phalanx; humerus *; anteriorphalanx *; metatarsal *; posterior phalanx *	upper molar row distal radius; metacarpal; two astragali; two cubonaviculars;	two metacarpals two metatarsals complete manus ** fragmentary ulna ** distal radius ** fragmentary humerus **
Unit 1	one worn DP4				

**Table 7:** Distribution of *Samotherium boissieri*, *Bohlinia attica*, and *Tragoportax rugosifrons*, data from https://nowdatabase.org/now/database/ by The NOW Community/CC BY 4.0, accessed 4 May 2025; *: several paleontological sites in the locality.

Locality	Country	Age (Ma)	*Samotherium* *boissieri*	*Bohlinia* *attica*	*Tragoportax* *rugosifrons*
Azmaka	Bulgaria	7.2		X	
Gorna Susica	Bulgaria	8.3–7.285		X	
Hadjidimovo	Bulgaria	7.6–7.1		X	X
Kalimantsi	Bulgaria	7.6–7.1		X	
Krodimovo	Bulgaria	7.6–7.1		X	
Strumyani	Bulgaria	7.6–7.1		X	X
Kirokuçuk	N Macedonia	8.9–5.3		X	
Dolni Disan	N Macedonia	8.9–5.3		X	
Karaslari	N Macedonia	7.6–7.1		X	X
Ditiko	Greece	7.1–5.3		X	
Kerassia	Greece	8.9–7.1		X	
Nikiti	Greece	9.9–8.9		X	
Pikermi *	Greece	7.454–7.1		X	
Pyrgos Vassilissis	Greece	7.212–7.14		X	
Ravin de la Pluie	Greece	9.426–9.311		X	
Ravin Zouaves *	Greece	9.9–8.1		X	
Samos *	Greece	8.9–5.3	X		X
Vathylakkos *	Greece	7.489–7.454		X	
Corakyerler	Turkey	8.9–7.6	X	X	
Esendere	Turkey	8.9–7.6		X	
Küçükçekmece	Turkey	8.9–5.3		X	
Gülpinar	Turkey	7.6–7.1	X		
Karain	Turkey	8.9–7.1	X		
Kavakdere	Turkey	8.254–8.108	X		
Sinap *	Turkey	11.2–7.1		X	
Injana	Iraq	8.9–7.6	X	X	
Maragheh *	Iran	8.9–7.1		X	X
Novo-Elizavetovka	Ukraine	8–9–7.1	X		
Taraklia	Moldova	7.6–7.1	X		X

## Data Availability

The original contributions presented in this study are included in the article. Further inquiries can be directed to the corresponding author.

## Abbreviations

The following abbreviations are used in this manuscript:

MuRi	City Museum of Ricadi, Via Roma, 12, 89866 Santa Domenica, Vibo Valentia, Italy
SiMU	Museal System of the University of Calabria, Section of Palaeontology, Via Pietro Bucci 87036 Rende, Cosenza, Italy
MuMe	“Museo della Memoria”, Via Trento 2, Parghelia, Vibo Valentia, Italy
GPT	“Gruppo Paleontologico Tropeano”, last address at the “Civico Museo del Mare di Tropea”, vico Ospedale n. 1 89861—Tropea (VV) Vibo Valentia, Italy
